# Depression and anxiety and their association with healthcare utilization in pediatric lupus and mixed connective tissue disease patients: a cross-sectional study

**DOI:** 10.1186/1546-0096-12-42

**Published:** 2014-09-10

**Authors:** Andrea Knight, Pamela Weiss, Knashawn Morales, Marsha Gerdes, Allyson Gutstein, Michelle Vickery, Ron Keren

**Affiliations:** Division of Rheumatology, Children’s Hospital of Philadelphia, 3405 Civic Center Blvd, Philadelphia, PA 19104 USA; Center for Pediatric Clinical Effectiveness, Children’s Hospital of Philadelphia, 3535 Market St, 15th Flr, Philadelphia, PA 19104 USA; Children’s Hospital of Philadelphia PolicyLab, Children’s Hospital of Philadelphia, 3535 Market St, 15th Flr, Philadelphia, PA 19104 USA; Center for Clinical Epidemiology & Biostatistics, University of Pennsylvania, 8th Flr Blockley Hall, 423 Guardian Drive, Philadelphia, PA 19104 USA; Department of Child and Adolescent Psychiatry and Behavioral Science, Children’s Hospital of Philadelphia, 3401 Civic Center Blvd, Philadelphia, PA 19104 USA; Division of General Pediatrics, Children’s Hospital of Philadelphia, 3401 Civic Center Blvd, Philadelphia, PA 19104 USA; Florida International University, Herbert Wertheim College of Medicine, 11200 SW 8th Street, AHC2, Miami, FL 33199 USA; Children’s Hospital of Philadelphia, 3535 Market St, Ste 1527, Philadelphia, PA 19104 USA

**Keywords:** Systemic lupus erythematosus, Pediatric, Depression, Anxiety, Suicide, Healthcare utilization

## Abstract

**Background:**

Depression and anxiety adversely affects outcomes in systemic lupus erythematosus (SLE) and healthcare utilization is high for pediatric SLE. We aimed to characterize the prevalence of depression and anxiety in pediatric SLE, and their association with healthcare utilization.

**Methods:**

We conducted a cross-sectional analysis of pediatric SLE and mixed connective tissue disease (MCTD) subjects and healthy controls aged 8 years and above. We used the Patient Health Questionnaire 9 (PHQ-9) and the Screen for Childhood Anxiety Related Disorders (SCARED) to identify depression, suicidal ideation and anxiety symptoms, respectively. We compared symptom prevalence in SLE/MCTD and healthy subjects using logistic regression. For SLE/MCTD subjects, we calculated the rate of annual outpatient visits [rheumatology/nephrology, primary care provider (PCP) and emergency department], hospitalizations and rheumatology/nephrology telephone consultations in the preceding year. We compared these outcomes in those with and without depression and anxiety using negative binomial regression.

**Results:**

We identified depression symptoms in 10 (20%) SLE/MCTD and 4 (8%) healthy subjects, representing a trend towards increased prevalence in unadjusted analysis (OR = 2.9, 95% CI 0.8-9.9, p = 0.09). Adjusted analysis did not show a significant difference; however, non-white race was a statistically significant independent risk factor for depression symptoms compared to white race (OR = 5.4, 95% CI 1.1-27.2, p = 0.04). We identified anxiety symptoms in 11 (22%) SLE/MCTD and 13 (26%) healthy subjects, which was not statistically different. Suicidal ideation was present in 7 (14%) SLE/MCTD and 2 (4%) healthy subjects, which was a statistically significant difference (OR = 5.4, 95% CI 1.02-28.3, p = 0.047). Of the 34% of SLE/MCTD subjects with any symptoms, only 24% had previous mental health care. Those with depression symptoms had a statistically significant lower rate of visits to the PCP (IRR = 0.38, 95% CI 0.19-0.76, p < 0.001). Anxiety symptoms were not associated with the healthcare utilization outcomes.

**Conclusions:**

Depression and anxiety symptoms were prevalent, and suicidal ideation significantly more common in SLE/MCTD than in healthy subjects. Non-white race was an independent risk factor for depression. Despite prevalent symptoms, there were poor rates of prior mental health treatment, and less frequent PCP visits among those with depression symptoms. Further investigation of barriers to mental health care and interventional strategies for symptomatic youth with SLE/MCTD is needed.

## Background

Systemic lupus erythematosus (SLE) and the SLE-like syndrome of mixed connective tissue disease (MCTD) are chronic autoimmune diseases characterized by systemic multi-organ inflammation, with significant morbidity and mortality occurring due to the disease and its treatment. Pediatric-onset SLE and MCTD represent a substantial portion of the overall disease burden, with approximately 15-20% of cases beginning in childhood at a median age of onset of 12–13 years [1]. Thus pediatric SLE and MCTD patients typically experience disease onset in early adolescence, a critical time in their psychosocial development of independence, self-identity and skills necessary for successful transition to adult roles and relationships [2]. Several studies indicate a high prevalence of depression in pediatric SLE [3–10], but fewer studies have examined anxiety which is often co-morbid with depression [11]. In pediatric chronic disease such as asthma, diabetes and inflammatory bowel disease, depression and anxiety have been shown to result in poorer disease control, quality of life, school performance and transition to adult care [12–15]. In adults with SLE, depression and anxiety are associated with increased disease activity, decreased medication adherence, and work productivity [16–19]. These disorders are therefore likely to have significant negative impacts on outcomes for pediatric SLE and MCTD patients if not addressed.

Healthcare utilization is high for children and adolescents with SLE [16, 17, 20] and represents an important outcome in this group. Frequent medical visits for management of SLE have potential for adverse effects on school and family functioning [21]. Healthcare utilization for patients with SLE also comprises a significant economic burden on national health care resources [22]. For example, the mean annual direct cost of caring for a pediatric SLE patient, including subspecialist and primary care visits, emergency department (ED) visits and hospitalizations, is approximately $15,000, compared to $1000 for pediatric asthma and $10,000 for inflammatory bowel disease [22]. Given that depression and poorer mental health are associated with increased healthcare utilization and costs in adults with SLE [16, 17, 20] as well as children with other chronic conditions [23, 24], early identification and treatment of depression and anxiety in children and adolescents with SLE or MCTD may lead to decreased healthcare utilization. There are no studies to our knowledge, however, specifically looking at the effects of depression and anxiety on healthcare utilization in pediatric SLE and MCTD.

We used a cross-sectional design to examine the prevalence of depression and anxiety and the association of these disorders with several types of healthcare utilization in a cohort of pediatric-onset SLE and MCTD patients. Specifically we aimed to characterize: 1) the prevalence of depression and anxiety in pediatric SLE and MCTD patients compared to healthy children and adolescents; and 2) the association of depression and anxiety with annual outpatient visits, hospitalizations, and telephone consultations to rheumatology/nephrology providers for SLE and MCTD patients. We hypothesized that pediatric SLE and MCTD patients would have a higher prevalence of depression and anxiety than their healthy peers, and that SLE and MCTD patients with depression and/or anxiety would have higher healthcare utilization than those without these mental health problems.

## Methods

### Study design, setting & participants

We performed a cross-sectional analysis on a cohort of pediatric SLE and MCTD subjects and a cohort of healthy children and adolescents at The Children’s Hospital of Philadelphia (CHOP). SLE and MCTD subjects were consecutively recruited during routine outpatient visits to rheumatology and nephrology between June 2012 and May 2013. SLE subjects had a diagnosis of pediatric-onset SLE (SLE diagnosed prior to the 18^th^ birthday when > =4 of 11 SLE classification criteria [25] were fulfilled). MCTD subjects had a diagnosis of MCTD if they met either Kahn’s or Alarcon-Segovia’s criteria for MCTD [26] prior to the 18^th^ birthday. Exclusion criteria were as follows: age less than 8 years; limited English proficiency, cognitive or communication deficit precluding questionnaire completion; isolated cutaneous lupus. Of 67 eligible subjects approached, 50 (75%) consented to the study. In order to maximally capture healthcare utilization, we restricted our utilization analysis to 42 patients with a minimum disease duration of 6 months from the date of diagnosis, and at least 2 visits to outpatient rheumatology or nephrology clinic within the 12 months prior to the study. Healthy participants were consecutively recruited during routine outpatient visits to CHOP primary care between October 2013 and May 2014. Exclusion criteria were as follows: age less than 8 years; limited English proficiency; cognitive or communication deficit precluding questionnaire completion; current use of steroids; diagnosis with a chronic medical condition including diabetes, irritable bowel disease, persistent ashma, cerebral palsy, sickle cell anemia, cystic fibrosis, cancer, HIV/AIDS, congenital heart disease, any other chronic autoimmune, hematologic, pulmonary, renal, gastrointestinal, neurological, or musculoskeletal condition. Of 112 eligible patients approached, 50 (45%) consented to the study. Informed consent was obtained from all participants and approval from the Institutional Review Board at the Children's Hospital of Philadelphia was obtained prior to initiation of the study.

### Measures of exposure: depression and anxiety

We screened for depression symptoms in the SLE/MCTD and healthy control cohorts using the Patient Health Questionnaire −9 (PHQ-9), a 9-item depression screening module based on the Diagnostic and Statistical Manual IV (DSM IV) criteria for major depression [27], which has been validated in the general adolescent population [28]. Each symptom criteria assesses feelings over the previous 2 weeks, and is scored from 0 (not at all) up to 3 (nearly every day), giving a total score range from 0 to 27. A tenth item uses the same scoring schema to assess functional impairment for those patients scoring > =1 on any of the 9 symptom criteria. A categorical scoring algorithm as follows indicates a positive screen: total score > =5, in addition to the first 2 items scored as > =2, and the tenth item scored as > =1. Positive screens with scores of 5–9, 10–14, 15–19 and 20–27 represent mild, moderate, moderately severe and severe depression symptoms, respectively. Suicide risk was assessed via item 9 on the PHQ-9 questionnaire. Scores > =1 for this item were considered indicative of suicidal ideation, and were also considered a positive screen for depression symptoms regardless of total PHQ-9 score; however, these positive screens were not categorized for depression severity if the total PHQ-9 was <5.

We screened for anxiety symptoms in the SLE/MCTD and healthy control cohorts using the Screen for Childhood Anxiety Related Disorders (SCARED), a 41-item anxiety screening tool which has been validated in outpatient children and adolescents [29]. The items ask about feelings in the previous 3 months, focusing on five factors: somatic/panic, general anxiety, separation anxiety, and social phobia. Each item has 3 possible answers: “not true or hardly ever true” (score of 0), “somewhat true or sometimes true” (score of 1), and “very true or often true” (score of 2). Item scores were summed, with a total > =25 indicating a positive screen for the presence of anxiety symptoms.

Depression and anxiety screening was performed using REDCap (Research Electronic Data Capture) electronic survey and data capture tools hosted at CHOP [30]. Upon identification of depression or anxiety symptoms, an educational handout was provided to the family with mental health care referral information. Identified suicide risk was addressed with a suicide prevention protocol consisting of immediate direct questioning of suicidal intent, plan or attempt within the prior week; endorsement of any of these prompted development of a safety plan and urgent referral for immediate psychology/psychiatry evaluation.

### Measures of outcome: healthcare utilization

For the SLE and MCTD subjects, we calculated utilization rates for the primary outcome of annual outpatient visits, and hospitalizations and telephone consultations as secondary outcomes. Outpatient visits were a composite of CHOP rheumatology, CHOP nephrology, primary care provider (PCP) and emergency department (ED) visits to any healthcare institution. Hospitalizations included those at any healthcare institution for any reason. Telephone consultations included phone calls to the CHOP rheumatology or nephrology offices, initiated by the patients or caregivers, regarding disease-related issues. We included these telephone consultations because they are a potentially important point of provider contact for clinical assessment, as well as an often unmeasured component of provider services. We did not include medication refill requests. For those patients with disease duration > =1 year, we observed healthcare use over a utilization period of one year preceding the study visit. For those with disease duration <1 year, we used a utilization period starting after the diagnosis date and extending to the time of study visit, excluding healthcare services used at the time of diagnosis. Electronic REDCap surveys were administered to participant parents/legal guardians at the time of enrollment to capture self-reported utilization data. Manual chart review of both CHOP and outside provider records was used to verify the reported utilization. The rate of outpatient visits, hospitalizations and telephone consultations per person-year were obtained by dividing the number of services used by the number of person-years of observation in the utilization period.

### Demographic variables and disease characteristics

The following variables were collected by survey of the parents/legal guardians for SLE, MCTD and healthy control subjects: age, sex, race/ethnicity, highest household education level, annual household income, quality of life (QOL) and subject mental health history. Race/ethnicity was categorized into 3 mutually exclusive groups: white, black and other (includes Hispanic, Asian/Pacific Islander, Native American and other). Highest household education level was categorized as either 1) less than college (includes incomplete college or less) or 2) college (includes completed associate, bachelors or advanced degree) and above. Annual household income was categorized into 3 groups according to the US national poverty guidelines for a household maximum of 8 people for the years of study [31]: less than $40000, $40000 and above, and prefer not to answer. QOL was measured using the Pediatric Rheumatology Quality of Life Scale (PRQL), a concise 10-item instrument measuring the core dimensions of physical and psychological health, with scores ranging 0–30 (higher score indicates poor QOL), which has been validated in European and US cohorts of pediatric rheumatology patients [32, 33]. Mental health history (Y/N) included any of the following: a previous psychiatric diagnosis, use of psychiatric medications or previous care by a psychiatrist or psychologist in the preceding 12 months.

The following variables were abstracted from electronic medical records of SLE and MCTD subjects: primary insurance type, disease type, disease duration, disease manifestations, immunosuppressive medications, physician global assessment score (PGA), SLE disease activity and pain score. Primary insurance was categorized as Medicaid/Medicare, private or other (includes self-pay and no charge). Disease type was categorized as SLE or MCTD based on disease criteria as detailed above. Disease manifestations included the following, as defined by the American College of Rheumatology (ACR) SLE classification criteria and documented by a rheumatology or nephrology provider at any point in the disease course: cytopenia, arthritis, rash, nephritis, serositis, central nervous system (CNS) disease. Antiphospholipid syndrome (APS) status was determined according to the 2006 APS International Consensus Classification Criteria [34]. Immunosuppressive medications included: current use of hydroxychloroquine, methotrexate, mycophenolate mofetil, azathioprine, glucocorticoids and aspirin; and history of rituximab, cyclophosphamide or dialysis treatment. Current glucocorticoid use was categorized into 3 groups: 1) no use 2) low-dose (<10 mg daily Prednisone equivalent) and 3) high-dose (> = 10 mg daily Prednisone equivalent). The PGA was assessed at the study visit (baseline) and for all visits to rheumatology in the utilization period, using a Likert scale ranging from 0 to 10, with higher scores indicating higher disease activity. SLE disease activity was measured for SLE patients at the study visit (baseline) and for all visits to rheumatology or nephrology in the utilization period, using the Safety of Estrogens in Lupus Erythematosus National Assessment (SELENA) modification of the SLE disease activity index (SLEDAI) [35, 36]. Pain score was assessed at the time of study visit by participant self-report on a Likert scale of 0 to 10, with higher scores indicating higher pain levels. Physical functioning of SLE and MCTD subjects was also assessed by survey of the parents/legal guardians using the Child Health Assessment Questionnaire (CHAQ), a validated 30-item instrument that measures 8 functional ability domains and calculates a disability index ranging from 0 to 3 (higher index indicates more disability) [37, 38].

### Statistical analyses

We performed all analyses and statistical comparisons using Stata 13 (Stata Corp., College Station, TX). Means and standard deviations (SD), medians and interquartile ranges (IQR), and frequencies were calculated for demographic and disease-related variables in the healthy control, full and restricted SLE/MCTD (for utilization analysis) cohorts. The presence of depression and anxiety symptoms were analyzed as binary covariates according to the above criteria for positive screens on the PHQ-9 and SCARED, respectively. We calculated prevalence rates for depression and anxiety in the SLE/MCTD and healthy control cohorts, and compared the rates between the full SLE/MCTD and healthy control cohorts using separate logistic regression analyses for depression and anxiety. We then conducted separate multivariable logistic regression analyses for depression and anxiety adjusting for demographic covariates. The race/ethnicity variable consisted of 2 categories (white and non-white) and the household income variable consisted of 2 categories (<$40000 and > =$40000, otherwise missing) for the multivariable analyses. We screened for potential confounders and effect modifiers by analyzing pairwise associations between the covariates and the exposure and outcome variables. To avoid overfitting, we included in the multivariate models only covariates with statistically significant pairwise associations (p < 0.2) with both the exposure and outcome variables. We performed a secondary analysis of the prevalence rate for suicidal ideation. The presence of suicidal ideation was analyzed as binary covariate according to a positive suicide risk on the PHQ-9 as described above. We calculated the prevalence rate for suicidal ideation in the full SLE/MCTD and healthy control cohorts, and compared the rates between cohorts using logistic regression analysis. We then conducted separate multivariable logistic regression analyses for suicidal ideation adjusting for demographic covariates as described above.

For the SLE/MCTD cohort, we calculated healthcare utilization rates per person-year with 95% confidence intervals (CI), and examined the association of these rates with depression and anxiety using separate negative binomial regression models. We conducted separate multivariable negative binomial regression analyses for depression and anxiety, adjusting for demographic and disease characteristic covariates. We screened for potential confounders and effect modifiers by analyzing pairwise associations between the covariates and the exposure and outcome variables. To avoid overfitting, we included in the multivariable model only covariates with statistically significant pairwise associations (p < 0.2) with both the exposure and outcome variables. We calculated standardized utilization rates from the multivariable analyses and compared them according to depression and anxiety status. We also performed secondary analyses to evaluate the outcomes by outpatient visit type (rheumatology/nephrology, PCP and ED). Due to the exploratory nature of these secondary analyses, correction for multiple testing was not applied. Throughout the analysis, all testing was 2-sided, with a threshold for statistical significance of p < 0.05.

## Results

### Demographics & disease characteristics

We enrolled 50 SLE/MCTD subjects, of which 43 had SLE and 7 had MCTD. Subject ages ranged from 9 to 20 with a median of 16.5 years (IQR 13.0,17.9). Females comprised 43 (86%) of the subjects. Twenty-three (46%) were white, 18 (36%) were black and 9 (18%) were of “other” race/ethnicity. We enrolled 50 healthy controls, whose ages ranged from 9 to 21 with a median of 13.4 years (IQR 11.4, 15.2). Females comprised 30 (60%) of the subjects. Thirteen (26%) were white, 26 (52%) were black and 11 (22%) were of “other” race/ethnicity. Additional demographic characteristics are shown in Table 1. For the full SLE/MCTD cohort the most frequent disease manifestations were cytopenia (84%), arthritis (74%) and rash (42%). Current immunosuppressive treatment included mycophenolate mofetil in 25 (50%) and glucorticoids in 34 (68%). The median physican PGA over the utilization period was 0.5 (IQR 0,1), and the median SLEDAI score was 2 (IQR 0,4). Additional disease characteristics for the full and utilization SLE/MCTD cohorts are presented in Table 1.

**Table 1:** **Subject demographic & disease characteristics**

	Healthy controls N = 50	Full SLE/MCTD Cohort N = 50	Utilization SLE/MCTD Cohort N = 42
**Age in years, median (IQR)**	13.4 (11.4,15.2)	16.5 (13.0,17.9)	16.9 (13.0,18.0)
**Female, N (%)**	30 (60)	43 (86)	38 (90)
**Race/Ethnicity, N (%)**			
**Black**	26 (52)	18 (36)	15 (36)
**White**	13 (26)	23 (46)	21 (50)
**Other**	11 (22)	9 (18)	6 (14)
**Highest Household Education, N (%)**			
**Less than college**	12 (24)	9 (18)	8 (19)
**College and above**	38 (76)	41 (82)	34 (81)
**Annual Household Income**			
**<$40000**	12 (24)	10 (20)	7 (17)
**$40000 and above**	34 (68)	30 (60)	25 (60)
**Prefer not answer**	4 (8)	10 (20)	10 (24)
**Insurance (N,%)**	-		
**Medicaid**		20 (40)	17 (40)
**Private**		30 (60)	25 (60)
**PRQL, median (IQR)**	2.5 (1,5)	1 (0, 4)	1 (0,4)
**Diagnosis, N (%)**	-		
**SLE**		43 (86)	37 (88)
**MCTD**		7 (14)	5 (12)
**Disease Duration in months, median (IQR)**	-	23 (11,50)	35 (16, 57)
**Disease Manifestations, N (%)**	-		
**Cytopenia**		42 (84)	37 (88)
**Arthritis**		37 (74)	30 (71)
**Rash**		21 (42)	21 (50)
**Nephritis**		6 (12)	4 (10)
**Serositis**		6 (12)	6 (14)
**CNS disease**		3 (6)	3 (7)
**APS (lab only)**		7 (14)	7 (17)
**APS (lab and clinical)**		3 (6)	3 (7)
**Medications/Treatments, N (%)**	-		
**Hydroxychloroquine**		49 (98)	41 (98)
**Methotrexate**		11 (22)	9 (21)
**Mycophenolate Mofetil**		25 (50)	20 (48)
**Azathioprine**		2 (4)	2 (5)
Glucocorticoids*			
**None**		16 (32)	15 (36)
**Low-dose**		28 (56)	25 (59)
**High-dose**		6 (12)	2 (5)
**Aspirin**		19 (38)	17 (40)
**History of rituximab**		7 (14)	7 (17)
**History of cyclophosphamide**		8 (16)	7 (17)
**History of dialysis**		1 (2)	0 (0)
Baseline SLEDAI**, median (IQR)	-	2 (0, 4)	2 (0, 4)
SLEDAI in past year**, median (IQR)	-	2 (0, 4)	2 (0, 4)
Baseline PGA**, median (IQR)	-	0 (0, 1)	0 (0,1)
**PGA in past year, median (IQR)**	-	0.5 (0, 1.5)	0.5 (0,1)
Pain Score**, median (IQR)	-	0 (0, 0)	0 (0,0)
CHAQ Score**, median (IQR)	-	0 (0, 0)	0 (0,0)

Demographic and disease characteristics are presented for the healthy control and SLE/MCTD cohorts. *Low-dose glucocorticoid is <10 mg and high-dose is > =10 mg daily Prednisone equivalent. **Missing data is as follows: PRQL in 11 subjects; baseline SLEDAI in 4 SLE subjects and median SLEDAI in past year in 1 SLE subjects due to unavailability of laboratory data; baseline PGA in 1 subject; CHAQ in 10 subjects; pain score in 6 subjects.

### Depression & anxiety prevalence

Depression and/or anxiety symptoms were identified in 17 subjects (34%) in the full SLE/MCTD cohort and 13 (26%) in the healthy control cohort (Table 2). We found a trend towards increased prevalence of depression symptoms in 10 (20%) of SLE/MCTD subjects compared to 4 (8%) of the healthy controls in the unadjusted analysis (OR = 2.9, 95% CI 0.8-9.9, p = 0.09). After adjusting for the confounders of age, sex and race, the difference in depression symptom prevalence between SLE/MCTD subjects and controls was not statistically significant (OR = 2.7, 95% CI 0.7-10.2, p = 0.15). However, non-white race was a statistically significant independent risk factor for depression symptoms compared to white race in the adjusted model (OR = 5.4, 95% CI 1.1-27.2, p = 0.04). Anxiety symptoms were identified in 11 (22%) SLE/MCTD subjects and 13 (26%) healthy controls, which did not represent a statistically significant difference in unadjusted analysis (OR = 0.8, 95% CI 0.3-2.0, p = 0.64). Adjusting for the confounder of sex in the multivariable analysis also did not show a statistically significant difference in anxiety prevalence. Of the 10 SLE/MCTD subjects with depression symptoms, 7 (70%) had suicidal ideation, representing 14% of the full SLE/MCTD cohort. In the healthy controls, 2 of the 4 (50%) with depression symptoms had suicidal ideation, representing 4% of the healthy control cohort. In unadjusted analysis, we found a trend towards increased prevalence of suicidal ideation in the SLE/MCTD cohort compared to the healthy controls (OR = 3.9, 95% CI 0.8-19.8, p = 0.1). After adjusting for the confounder of race this difference was statistically significant (OR = 5.4, 95% CI 1.02-28.3, p = 0.047). None of the SLE/MCTD subjects and one of the healthy controls required urgent referral for suicidal ideation. Of the subjects who screened positive for any symptom (depression, anxiety and/or suicidal ideation), 4 (24%) SLE/MCTD subjects and 1 (8%) healthy control reported previous mental health care. Additional mental health characteristics are presented in Table 2.

**Table 2:** **Subject mental health characteristics: depression, suicidal ideation & anxiety prevalence**

	Healthy N = 50	SLE/MCTD N = 50	OR (95% CI) p-value	
			Unadjusted	Adjusted
**Depression, N (%)**	4 (8)	10 (20)	2.9 (0.8-9.9) p = 0.09	2.7 (0.7-10.2) p = 0.15
Anxiety*, N (%)	13 (26)	11 (22)	0.8 (0.3-2.0) p = 0.64	0.7 (0.2-1.7) p = 0.38
**Suicidal Ideation, N (%)**	2 (4)	7 (14)	3.9 (0.8-19.8) 0.10	5.4 (1.02-28.3) p = 0.047
Any symptom (depression and/or anxiety*), N (%)	13 (26)	17 (34)	-	-
**PHQ-9 score in depressed, mean (SD)**	14 (6)	11 (5)	-	-
**Depression Symptom Severity, N (%)**				
**Mild**	0 (0)	5 (10)		
**Moderate**	2 (4)	2 (4)		
**Moderately Severe**	0 (0)	2 (4)		
**Severe**	1 ((2)	1 (2)		
**SCARED score in anxious, mean (SD)**	33 (8)	34 (7)	-	-
**Co-morbid depression & anxiety, N (%)**	3 (6)	3 (6)	-	-
**Mental Health History (N,%)**	5 (10)	5 (10)	-	-
**Mental Health History in those with any symptom, N (%)**	1 (8)	4 (24)	-	-

The prevalence of depression, anxiety and suicidal ideation symptoms, and other mental health characteristics of the healthy control and full SLE/MCTD cohorts are shown. The logistic regression model for depression was adjusted for age, sex and race. The logistic regression model for anxiety was adjusted for sex. The logistic regression model for suicidal ideation was adjusted for race. Depression symptom severity was categorized according to the following PHQ-9 score ranges: mild 5–9; moderate 10–14; moderately severe 15–19; and severe 20–27. Two subjects screened positive for suicidal ideation and therefore depression, but were not categorized for depression. *Missing data is as follows: SCARED in 1 SLE/MCTD patient (2%).

### Healthcare utilization

**Figure 1:**
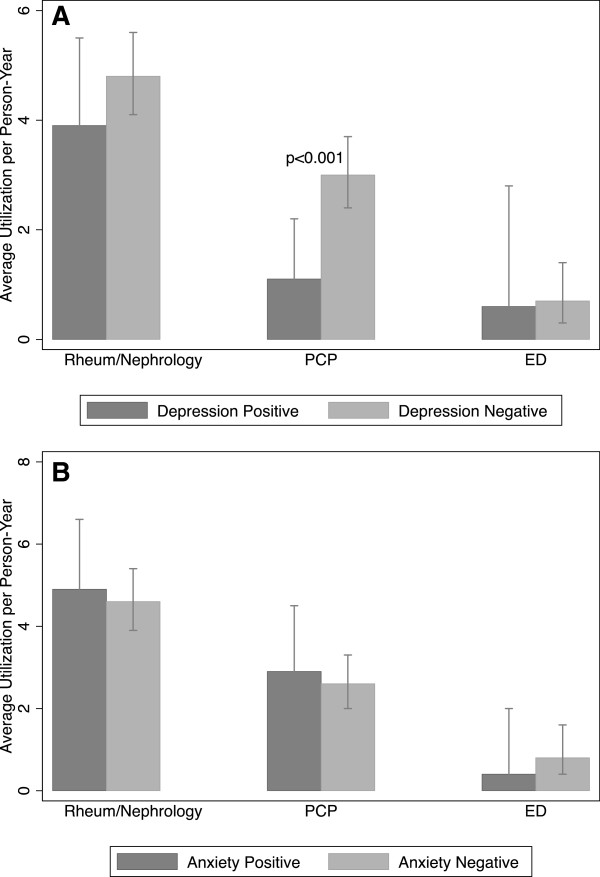
**Unadjusted utilization estimates by outpatient visit type according to depression status. A** Unadjusted utilization estimates by outpatient visit type according to depression status are shown. Visit types include rheumatology/nephrology, primary care provider (PCP) and emergency department (ED). Patients with depressive symptoms were statistically less likely to attend PCP visits than those without symptoms (IRR = 0.38, 95% CI 0.19-0.76, p < 0.001). Error bars represent 95% confidence intervals. **B** Unadjusted utilization estimates by outpatient visit type according to anxiety status are shown. Visit types include rheumatology/nephrology, primary care provider (PCP) and emergency department (ED). There were no significant differences in outpatient visit types according to anxiety status. Error bars represent 95% confidence intervals.

We analyzed healthcare utilization over a total of 41 person-years in the restricted SLE/MCTD cohort. As estimated from unadjusted negative binomial regression models, the overall outpatient visit rate per person-year was 7.9 (95% CI 6.9-9.1). These were comprised of an average number of visits per person-year of 4.3 (95% CI 3.7-5.0) to rheumatology, 0.4 (95%CI 0.06-2.7) to nephrology, 2.6 (95% CI 2.1-3.3) to primary care providers, and 0.7 (95% CI 0.4-1.3) to the emergency department. The average number of hospitalizations per person-year was 0.3 (95% CI (0.1-0.9) and phone consultations to rheumatology and/or nephrology was 1.7 (95% 1.2-2.5). In the regression analysis that included depression as the primary risk factor, we found that patients with depressive symptoms were significantly less likely to attend outpatient visits compared to those without depressive symptoms (IRR = 0.67, 95% CI 0.46-0.96, p = 0.03) (Table 3). Secondary analysis by outpatient visit type showed patients with depressive symptoms were statistically less likely to attend PCP visits than those without symptoms (IRR = 0.38, 95% CI 0.19-0.76, p < 0.001) (Figure 1A). There was no difference in the rate of rheumatology/nephrology or ED visits with regard to depression status. Depressive symptoms were also not associated with differences in hospitalizations or phone consultations. Multivariable analysis was not performed for the depression model because we did not identify potential confounders or effect modifiers for inclusion. In the regression analysis that included anxiety as the primary risk factor, we found no difference in the rate of outpatient visits (overall and by visit type), hospitalizations or phone consultations by anxiety status (Table 4, Figure 1B). For multivariable analysis, education was identified as a potential confounder and included in the regression models for all the utilization outcomes. We also found no difference in the rate of outpatient visits, hospitalizations or phone consultations by anxiety status in the multivariable models (Table 4).

**Table 3:** **Results of the unadjusted negative binomial regression analysis of healthcare utilization on depression**

Outcome	Average utilization per person-year, number (95% CI)		IRR (95% CI) p-value
	Positive depression	Negative depression	
**Outpatient visits**	5.7 (4.0-7.9)	8.5 (7.3-9.8)	0.67 (0.46-0.96) 0.03
**Hospitalizations**	0.1 (0.01-2.5)	0.3 (0.1-1.1)	0.38 (0.01-9.92) 0.56
**Phone consultations**	1.0 (0.4-2.5)	1.9 (1.3-2.8)	0.52 (0.19-1.41) 0.20

Estimates of healthcare utilization by depression status from unadjusted negative binomial regression analysis models are shown.

**Table 4:** **Results of the unadjusted and adjusted negative binomial regression analyses of healthcare utilization on anxiety**

Outcome	Unadjusted analysis			Adjusted analysis		
	Average utilization per person-year, number (95% CI)		IRR (95% CI) p-value	Average utilization per person-year, number (95% CI)		IRR (95% CI) p-value
	Positive anxiety	Negative anxiety		Positive anxiety	Negative anxiety	
**Outpatient visits**	8.2 (6.1-11.0)	7.9 (6.7-9.3)	1.03 (0.74-1.44) 0.84	11.4 (7.4-17.7)	10.0 (7.6-13.3)	1.14 (0.82-1.59) 0.45
**Hospitalizations**	0.2 (0.02-2.9)	0.3 (0.1-1.2)	0.70 (0.04-12.5) 0.81	5.8 (0.2-147.3)	1.1 (0.2-5.2)	5.1 (0.3-88.7) 0.26
**Phone consultations**	2.3 (1.2-4.7)	1.6 (1.1-2.4)	1.44 (0.64-3.22) 0.38	4.9 (1.7-14)	2.7 (1.4-5.3)	1.84 (0.8-4.1) 0.14

Estimates of healthcare utilization by anxiety status from negative binomial regression analysis models are shown. Multivariable analyses include education level as a covariate, and standardized estimates are presented for subjects with highest household education level of college and above.

## Discussion

This cross-sectional analysis of a cohort of SLE and MCTD children and adolescents and their healthy peers adds to the sparse literature on mental health in pediatric SLE. We present novel data on prevalence rates of depression, anxiety and suicidal ideation for children and adolescents with SLE/MCTD, as well as estimates of the association of depression and anxiety symptoms with healthcare utilization for this population. Depression and anxiety symptoms were prevalent, affecting 34% of our cohort, with depression symptoms in 20%, anxiety in 22% and suicidal ideation in 14%. These estimates are similar to previous studies of pediatric patients with SLE that indicate a prevalence of 15-55% for depression and 15-20% for anxiety [3–10]. The etiology of depression and anxiety in pediatric SLE and MCTD patients is likely complex and related several factors including: the psychological stress of dealing with a chronic illness during adolescence [39]; inflammatory central nervous system (CNS) pathology related to SLE [40]; direct steroid CNS effects [41] and indirect steroid-related somatic effects (eg. appearance concern due to weight gain, acne and striae) [42], as well as; hereditary and environmental factors [16, 18, 43].

In comparison to their healthy peers, there was a trend towards higher depression symptom prevalence in SLE/MCTD patients in unadjusted analysis; however, our adjusted analysis (for age, sex and race) did not show a significant difference. There was also no significant difference in anxiety symptom prevalence. These findings suggest that SLE and MCTD do not convey additional depression and anxiety risk from the disease itself. It is notable, however, that our SLE/MCTD cohort had a low occurrence of diagnosed CNS disease (6%) and low SLE disease activity. Although the relationship of depression and anxiety to SLE disease activity remains unclear in the literature [7, 9, 18, 40, 43–46], it is possible that our findings underestimate depression and anxiety prevalence in pediatric SLE populations with higher disease activity. Our results must be interpreted cautiously, however, as it is difficult to make causal associations and generalizable conclusions based on our cross-sectional analysis of a single cohort. We also did not evaluate additional potential risk factors such as steroid treatment, psychological stress, parental mental health and family stress in our limited study; inclusion of these covariates would likely be useful in larger studies. Nevertheless, the fact that depression and anxiety symptoms affected 34% of our cohort warrants the attention of pediatric rheumatologists to these common conditions which are likely to have an adverse impact on outcomes for pediatric SLE patients. Depression and anxiety are shown to result in poorer disease control, quality of life, school performance and transition to adult care in other pediatric chronic disease such as asthma, diabetes and inflammatory bowel disease [12–15]. Furthermore, adults with SLE and depression/anxiety have associated poorer medication adherence, increased disease activity and lower work productivity [16–19]. Thus focusing on identification and intervention of depression and anxiety in children and adolescents with SLE could result in earlier treatment and better patient-oriented outcomes.

Of significant concern is our finding of suicidal ideation in 14% of our SLE/MCTD subjects, which showed a statistically significant increased prevalence compared to 4% of the healthy controls. The literature is scarce for suicidal ideation in pediatric SLE. Nassi et al. reported suicidal ideation in up to 19% of their pediatric SLE patients [47]. Lim et al. reported suicidal ideation in 34% of 53 pediatric patients with neuropsychiatric SLE, and 20% of those with ideation attempted suicide [48]. In contrast, Kohut et al. reported no suicidal ideation in a cohort 38 children and adolescents with SLE, of which 26% had depression symptoms [9]. However, studies in adults with SLE show suicidal ideation is common in up to one-third of patients with and without neuropsychiatric SLE [49, 50], and is associated with higher disease activity, increased depression and anxiety severity and previous suicide attempts [51]. Although none of our SLE/MCTD subjects with suicidal ideation posed an immediate suicide risk, our results build on the data supporting a need for increased awareness and ability to identify and intervene for suicidal ideation in pediatric patients with SLE/MCTD.

Given the levels of depression, anxiety and suicidal ideation, it is concerning that only 24% of subjects in our cohort with any of these symptoms reported previous mental health care. There are several possible reasons occurring at the patient, caregiver, provider and healthcare system levels that may be contributing. At the patient level, non-white race was a statistically significant independent risk factor for depression symptoms compared to white race in our cohort. This is of particular importance for pediatric SLE patients who are a disproportionately non-white group [1]. The reason for this increased risk is unclear but may be related to known cultural stigma and socioeconomic barriers to diagnosing and managing depression in those of non-white race [52–54]. This has implications for ensuring appropriate depression identification and access to treatment of youth with SLE.

At the provider level, mild symptoms may be overlooked as part of normal adolescent development; however, persistent mild or “subthreshold” depression symptoms in adolescence are a predictor of development of major depression and suicidal behavior in later adulthood [55, 56] and should be evaluated. Despite the availability of validated screening tools and evidence-based therapies for depression and anxiety [57–60], pediatric rheumatologists may feel ill-equipped to address the mental health needs of children and adolescents with SLE/MCTD. Recent national efforts have established the role of PCPs in screening and intervention for adolescent depression as part of a collaborative care model [61], but rheumatologists are often the principal providers for SLE patients [62], and may be better-suited for this role. Our outpatient utilization data supports this as our SLE/MCTD cohort visited their rheumatologists almost twice as much as their PCPs, affording the most opportunity to pediatric rheumatologists for addressing their mental health needs. Our finding that SLE/MCTD patients with depression symptoms visited their PCPs approximately 60% less frequently than those without symptoms suggests a potentially wider differential between PCP and rheumatology contact in those with depression. One might speculate that depressed children and adolescents and potentially concurrently depressed caregivers might exhibit negative coping styles and social withdrawal [63], resulting in less PCP contact but preserved visits to rheumatologists as their principal providers. Potential system level barriers to the implementation of screening and intervention in pediatric rheumatology practice including costs, reduced clinic efficiency and lack of behavioral health resources, deserve further investigation. Additionally, other associations between depression/anxiety and other healthcare utilization outcomes may exist with implications for mental health care treatment, but our study may be underpowered to detect them in our limited sample. Our results therefore serve to generate hypotheses for larger studies and longitudinal investigation of the relationship between depression/anxiety and healthcare utilization for children and adolescents with SLE/MCTD. Nonetheless, our findings highlight the fact that there are no existing guidelines or standard practices for mental health screening and intervention of depression, anxiety and suicidal ideation for pediatric SLE and MCTD patients in the context of pediatric rheumatology care.

## Conclusions

Depression and anxiety symptoms were prevalent, and suicidal ideation significantly higher in this cohort pediatric SLE and MCTD patients compared to their healthy peers. Non-white race was an independent risk factor for depression symptoms with implications for access to mental health care for youth with SLE, who represent a disproportionately non-white group. Despite mental health symptoms affecting one third of the cohort, rates of prior mental health treatment were poor, and PCP visits were less frequent among those with depression symptoms. Our results suggest an important role for pediatric rheumatologists in addressing mental health in symptomatic youth with SLE/MCTD. There is a need for increased awareness and further investigation of the barriers to mental health care and strategies for improving the mental health of pediatric SLE and MCTD patients.

## Authors’ information

AK: MD, MSCE, Assistant Professor of Pediatrics, University of Pennsylvania, Perelman School of Medicine.

PW: MD, MSCE, Assistant Professor of Pediatrics, University of Pennsylvania, Perelman School of Medicine.

KM: ScD, Assistant Professor of Biostatistics, University of Pennsylvania, Perelman School of Medicine.

MG: PhD, Clinical Associate Professor of Pediatrics, University of Pennsylvania, Perelman School of Medicine.

AG: BA.

MV: BS, MPH.

RK: MD, MPH, Professor of Pediatrics, University of Pennsylvania, Perelman School of Medicine.

## Abbreviations

SLE: Systemic lupus erythematosus
MCTD: Mixed connective tissue disease
PHQ-9: Patient Health Questionnaire- 9
SCARED: Screen for Childhood Anxiety Related Disorders
IRR: Incidence rate ratio
PCP: Primary care provider
ED: Emergency department
CHOP: Children’s Hospital of Philadelphia
DSM IV: Diagnostic and Statistical Manual IV
REDCap: Research Electronic Data Capture
QOL: Quality of life
PRQL: Pediatric Rheumatology Quality of Life Scale
CHAQ: Child Health Assessment Questionnaire
PGA: Physician global assessment
ACR: American College of Rheumatology
CNS: Central nervous system
APS: Antiphospholipid syndrome
SELENA: Safety of Estrogens in Lupus Erythematosus National Assessment
SLEDAI: Systemic lupus erythematosus disease activity index
CI: Confidence interval
SD: Standard deviation
IQR: Interquartile range
US: United States.
